# Pre-Hospital Stroke Care beyond the MSU

**DOI:** 10.1007/s11910-024-01351-0

**Published:** 2024-06-22

**Authors:** Kian j. Röhrs, Heinrich Audebert

**Affiliations:** 1 Department of Neurology, Campus Benjamin Franklin, Charité-Universitätsmedizin Berlin, Corporate Member of Freie Universität Berlin and Humboldt-Universität Zu Berlin, Hindenburgdamm 30, 12203 Berlin, Germany; 2 Center for Stroke Research Berlin, Charité-Universitätsmedizin Berlin, Corporate Member of Freie Universität Berlin and Humboldt-Universität zu Berlin, Berlin, Germany

**Keywords:** Stroke, Mobile stroke unit, Biomarker, Telemedicine, Emergency medicine, Artificial intelligence

## Abstract

**Purpose of review:**

Mobile stroke units (MSU) have established a new, evidence-based treatment in prehospital stroke care, endorsed by current international guidelines and can facilitate pre-hospital research efforts. In addition, other novel pre-hospital modalities beyond the MSU are emerging. In this review, we will summarize existing evidence and outline future trajectories of prehospital stroke care & research on and off MSUs.

**Recent findings:**

The proof of MSUs' positive effect on patient outcomes is leading to their increased adoption in emergency medical services of many countries. Nevertheless, prehospital stroke care worldwide largely consists of regular ambulances. Advancements in portable technology for detecting neurocardiovascular diseases, telemedicine, AI and large-scale ultra-early biobanking have the potential to transform prehospital stroke care also beyond the MSU concept.

**Summary:**

The increasing implementation of telemedicine in emergency medical services is demonstrating beneficial effects in the pre-hospital setting. In synergy with telemedicine the exponential growth of AI-technology is already changing and will likely further transform pre-hospital stroke care in the future. Other promising areas include the development and validation of miniaturized portable devices for the pre-hospital detection of acute stroke. MSUs are enabling large-scale screening for ultra-early blood-based biomarkers, facilitating the differentiation between ischemia, hemorrhage, and stroke mimics. The development of suitable point-of-care tests for such biomarkers holds the potential to advance pre-hospital stroke care outside the MSU-concept. A multimodal approach of AI-supported telemedicine, portable devices and blood-based biomarkers appears to be an increasingly realistic scenario for improving prehospital stroke care in regular ambulances in the future.

## Introduction

Neurological disorders and among these acute neurovascular diseases like stroke are primary contributors to global disease burden [1]. This has led to the formulation of specific plans to improve stroke care by pan-national professional organizations and health care providers [2, 3]. The time-critical nature of acute stroke (reflected by the axiom “time is brain”) is continuously prompting researchers and clinicians to develop new and to optimize already-existing therapeutic strategies to maximize effectiveness by minimizing latency to treatment. One of the most recognizable advancements in the treatment of acute stroke within the last decade certainly was the development of mobile stroke-units (MSU). MSUs are ambulances equipped with a CT-scanner for brain imaging (including CT angiography), a point-of-care laboratory, a telemedical interface and a specialized medical team. MSUs therefore allow for the on-scene confirmation of stroke suspicion and ultra-early initiation of drug therapy for ischemic and hemorrhagic stroke as well as targeted and direct hospital transfer of patients in need for further endovascular or surgical treatment. Even though the number of MSU operators around the world is constantly increasing, prehospital stroke care to date still largely consists of regular ambulances. In this review, we summarize the current practice and outline future perspectives of prehospital stroke care and research on and off mobile stroke units.

## Past, Present & Future of Mobile Stroke Units

After the first conceptualization of the idea of a mobile stroke unit in 2003 [4] several subsequent clinical studies demonstrated that mobile stroke units improve metrics of stroke care (such as “onset to treatment-time”, rate of “golden hour” thrombolysis, rate of thrombolysis, triage to the required level of care) compared to treatment by regular ambulances [5–13]. Recently, two large, controlled studies showed improved functional outcomes after dispatch or use of MSU.

The prospective, controlled interventional trial spearheaded in Berlin (B_PROUD^1^) compared 749 patients who received MSU-treatment with 794 patients treated by a conventional ambulance with the primary outcome being the degree of disability as quantified by the modified Rankin Scale-Score (mRS) and a 3-tier disability scale at day 90 [14]. MSU-Treatment was associated with a favorable shift in the distribution of the mRS-Score, as well as survival and autonomy in daily living.

Conducted in several cities in the United States, the prospective, multicenter, cluster-controlled “BEST-MSU” Trial^2^ compared 617 patients with ischemic stroke eligible for rt-PA treatment who received care by a MSU with 430 patients treated by emergency medical services [15] with regard to the degree of disability (mRS) at day 90. Patients who had been treated by an MSU prehospitally had more favorable outcomes. 53.5% of the patients in the MSU group had a favorable mRS-Score of 0–1 compared to 45.5% in the EMS group.

Sequential meta-analyses conducted to evaluate the cumulative evidence showed that the employment of mobile stroke units is correlated with better outcomes, as denoted by mRS-score ranging from 0 to 2 (indicating no significant disability to slight disability). Furthermore, MSUs reduced the time from symptom onset to start of thrombolytic treatment by on average 30 min. Additionally, a higher proportion of patients received intravenous thrombolysis, both in general and specifically within the first 60 min after symptom onset ("golden hour”) [16]. The administration of thrombolysis within the first hour is associated with a duplication of the odds for excellent clinical outcome [17]. Additionally, the possibility of intracranial vascular imaging via on-site CT-angiography in severely affected individuals allows for the identification of large-vessel occlusions and direct transportation to hospitals with thrombectomy capability. The growing evidence concerning beneficial effects of mobile stroke units led to the first international guideline endorsing MSU-treatment to improve prehospital care of patients with suspected stroke [18]. In parallel, cost–benefit analyses have been conducted in several countries [19–23] and suggest—together with consecutive meta-analyses [24]—an acceptable cost-effectiveness of MSU-deployment. In view of the evidence now available on the benefits and cost-effectiveness of mobile stroke units, members of the Prehospital Stroke Treatment Organization [25] are working together with health insurers to develop an appropriate reimbursement strategy to allow for regular MSU use.

Apart from their role in patient care, MSUs have a great potential to further advance prehospital stroke care by serving as “prehospital research platforms”. The presence of a specialized team aboard most MSUs, together with point-of-care imaging and laboratory equipment creates a mobile research unit. The fact that these MSU teams are specifically sent to patients with suspected acute stroke, creates an outstanding potential to scientifically investigate the pathogenesis of acute ischemic and hemorrhagic stroke in a time window that had largely been inaccessible to science so far. Through standardized, collaborative and international research efforts by MSU operators around the world, MSUs will likely become an integral part of the future scientific landscape in acute neurology.

Nevertheless, regular ambulances are still the predominant way of prehospital stroke care worldwide. This is in part due to resource limitations in lower income countries and communities, but also related to geographic barriers making MSUs, at least currently, impractical in some low-population density settings with current dispatching strategies. In the subsequent section we will discuss emerging fields with great potential to improve prehospital stroke care already now or otherwise in the near future and most importantly outside of classical MSUs aboard regular ambulances. These innovations include telemedicine, use of artificial intelligence in emergency medical services, the development of new imaging techniques for stroke and the first-time possibility to implement large-scale, ultra-early biobanking to facilitate biomarker discovery.

## Telemedicine & Artificial Intelligence

Telemedicine has been used in acute stroke care for over more than two decades [26, 27] with its predominant use for supporting remote hospitals lacking onsite neurologists. Utilization of telemedicine had also been suggested for assessing stroke patients during out-of-hospital emergency care and guide prehospital treatment [28] but low bandwidth and unreliable mobile communication availability limited its applications until recent years. The advancements both in device technologies and mobile data transmission coverage have opened new opportunities. Telemedicine can be used at different stages of prehospital emergency care and may be complemented by artificial intelligence.

The widespread availability of smartphones with videotelephony capacity and other wearable devices might be a promising resource to improve stroke recognition at dispatcher level. A dispatcher able to visually assess a patient, could lead to improved dispatching especially of helicopters in remote areas or MSUs in rural and metropolitan areas. In a study conducted by Linderoth et al. in Copenhagen, Denmark the technical feasibility of videotelephony during dispatch procedures was demonstrated and led to a change in the dispatchers’ assessment of patient conditions in 51.1% of the cases [29]. Recent meta-analyses point towards a beneficial effect on patients outcomes, especially in the setting of prehospital cardiac arrest [30, 31] and current guidelines in this field recommend further scientific evaluation of the concept [32]. The current advent of artificial intelligence will likely also have a transformative effect within the field of emergency medicine in general and prehospital stroke care in particular. The most common method of assessing an emergency is still the speech-based exchange of information between the emergency caller and the dispatcher with a human review of a rule-based algorithm [33]. This makes it an excellent application for Large Language Model-based machine learning approaches and studies in this field show promising results [34, 35]. The possible applications are broad. They encompass real-time translation when language barriers are present, analysis of speech patterns and content to identify the correct diagnosis [36–38], optimizing navigation to and from the incident scene e.g. by preemptive traffic light control to prioritize emergency vehicles in areas with crowded intersections [39], analysis of health-data to guide diagnosis [40] and could potentially also result in preemptive models of dispatch by integrating personal health data via electronic health records and by inclusion of real-time GPS-data of emergency vehicles with regard to the forecasted demand–supply-ratio in a specific area (for further review see [37, 38]). In the field of stroke, several studies are currently investigating AI-guided EMS dispatching [43].

Video examination in camera-equipped non-spezialized ambulances is currently becoming increasingly available and image-based deep learning applications can be envisioned to help with the identification of patients with stroke also on regular ambulances [44, 45]. Large language model-based approaches are already being investigated for their feasibility to increase the detection rate of patients with stroke from paramedic reports [46]. Especially aboard regular ambulances, telemedicine has been shown to be a feasible approach to identify reperfusion candidates through teleneurological assessment. In a study conducted by Scott et al. in New Zealand, telemedical consultation was initiated when paramedics had the suspicion of stroke and the accuracy of remote neurological assessment as well as a stroke-score-based approach (prehospital acute stroke triage and assessment, PASTA) conducted by a paramedic was examined. A remote neurological examination through a video-audio connection was equal to an in-person neurological examination in the emergency department and showed a predictive accuracy of 80% with regard to the rate of administered reperfusion therapies [47]. Alternatives to this approach encompass specialized stroke training for paramedics to speed up the detection of patients with stroke. In a survey conducted by Melaika et al. 44.8% of paramedics perceived their prehospital stroke care knowledge as inadequate [48]. The frequent use of prehospital stroke scales like e.g. RACE, G-FAST or CG-FAST could also lead to better treatment metrics aboard regular ambulances, even though the limitations of these approaches concerning sensitivity and specificity are well known [49, 50]. Lastly, public health campaigns to further educate patients about stroke symptoms and the time-critical nature of the disease could lead to a changed help-seeking behavior with earlier consultation of emergency medical services [51].

## New Technologies

Additional strategies for enhancing the efficiency of prehospital care for stroke patients beyond CT-equipped MSU predominantly concentrate on the development of new and the refinement of existing technological approaches aimed at accurate identification of strokes, their subtypes (ischemic or hemorrhagic) and stroke-mimicking diseases (stroke-mimics) aboard regular ambulances. Recent advances in the field of *low-field MRI* scanners, which offer advantages like lower weight, reduced electrical needs and a permanent magnetic field, have led to the construction of mobile MRI devices [52, 53]. Concept studies of mobile point-of-care MRI devices within a telemedicine-equipped ambulance exist and show, that MRI images can be generated during active patient transportation [54]. It is unclear whether and how these advances can or need to be translated into a broader scale given the associated costs of development and the existing evidence for the utility of CT-equipped MSUs, even though certain scenarios might exist, in which an MRI-based approach could be useful (wake-up-stroke, stroke-mimics). Similar approaches trying to further miniaturize mobile CT scanners to reach geographically more challenging areas by land or air are already conceptualized [55, 56] and could complement existing concepts in this field [57].

The use of *transcranial doppler-sonography (TCD)* is the gold-standard for point-of-care vascular evaluation of patients with stroke in the hospital. Due to its small size, comparably low prize, easy portability and the possibility of repeated measurements, TCD has already been investigated prehospitally for the use of prehospital LVO detection (for further review see [58, 59]). Even though studies continue to underscore the excellent diagnostic metrics of non-contrast TCD [60] and new sonographic biomarkers are being evaluated [61], prehospital validation is largely missing and could be challenging to achieve, as performing and interpreting a TCD measurement reliably and swiftly in a prehospital emergency setting requires a correspondingly high level of examiner expertise. On the other hand, efforts made in *automated and contrast-enhanced ultrasound* examinations, show promising results. The SONAS device is a portable ultrasound device designed to function like and resemble a headset. Together with the application of ultrasound contrast very small pilot studies could demonstrate the principal feasibility of this approach for detection of LVO-associated disturbed brain perfusion [62, 63]. In other fields, automated TCD is also increasingly investigated. Clare et al. could show feasibility for detection of vasospasm with the NovaGuide robotic TCD system in patients with subarachnoid hemorrhage on a neurological ICU [64]. Prehospital evaluation though is largely lacking currently. Taken together prehospital, automated TCD has the principal feasibility to help in the identification of patients with large-vessel occlusions and transcranial color coded dopplersonography has the capability to detect deep intracerebral, supratentorial hematoma [65]. For the detection of superficial or infratentorial intracerebral hemorrhages though, other technological approaches are needed and already being developed.

The *near-infrared spectroscopy technology (NIRS)* uses near-infrared wavelength to measure the ratio of oxygenated and deoxygenated hemoglobin. It has the property of penetrating the scull and the brain surface up to 2.5cm and promises a high sensitivity for intracranial hematoma due to the presence of relatively more hemoglobin within the hematoma compared to healthy tissue. In traumatic brain injury, NIRS devices show acceptable diagnostic metrics [66]. For the detection of ischemic stroke evidence is currently inconsistent. While Kwon et al. could observe an association of lower hemoglobin oxygenation within the hypoperfused hemisphere (as assessed via CT-Perfusion) [67], Collette et al. could not observe changes of hemoglobin oxygenation in patients with LVO undergoing thrombectomy [68]. Limitations of the technology currently include the fact that only hematoma > 3,5cm in a superficial localization (max. 2,5cm from the skull) can be detected reliably [58, 69].

Different concepts of *microwave-based devices* for stroke detection exist and could show promising detection rates in early studies [70–73]. Several devices like the Strokefinder [74, 75] are developed in the private sector and are currently tested within the acute setting of stroke [76]. Prehospital validation is lacking.

Other portable approaches include *Volumetric Impedance Phase-Shift Spectroscopy (VIPS)* which is based on the so-called magnetic induction tomography and measures stroke-associated changes of the electrical properties of brain tissue. Trials show promising results concerning the differentiation of stroke from stroke-mimicking diseases [77] but prehospital validation is lacking.

Preclinical studies showed that *Laser speckle contrast imaging* can be used to measure cerebral blood flow in mice [78]. Laser Speckle Contrast Imaging is a non-invasive optical method with high temporal and spatial resolution that relies on the phenomenon known as "laser speckle," which occurs when a laser beam illuminates a surface (such as tissue) and creates a random interference pattern. Movement of particles like red blood cells within the tissue then create fluctuations in the speckle pattern, which can be measured. Feasibility was recently shown in humans for a commercial device [79], which has already been tested in a cohort of patients with suspected stroke for LVO detection. Favilla et al. observed a 79% sensitivity and 84% specificity for the detection of LVO [80]. Prehospital validation is currently lacking.

Finally attempts to utilize *EEG* measurements to detect stroke-related changes of cortical electrical activity have been made mainly for the detection of LVO. Within the ELECTRA-STROKE trial van Stigt et al. observed a sensitivity 80% at a specificity of 93% for LVO-detection [81]. Translation into a prehospital setting seems difficult though, due to poor EEG quality obtained within a more acute setting (e.g. due to motion artifacts). In addition, EEG can only differentiate functioning versus non-functioning brain tissue, a distinction between ischemic and hemorrhagic stroke appears to be technically difficult to achieve.

Taken together, several emerging technologies for the detection of stroke currently exist, but translation into a prehospital setting aboard regular ambulances is still in its early steps of development and validation.

## Prehospital Biomarkers

Blood based biomarkers for stroke detection and differentiation between ischemic and hemorrhagic stroke could be used for prehospital stroke care like point-of-care Troponin-T-assessment in acute chest pain. Several smaller-scale clinical trials have already investigated a number of biomarkers in the prehospital setting of stroke. In a first trial aboard MSUs GFAP (glial fibrillary acidic protein) could be identified as a potential biomarker with high specificity for intracerebral hematoma [82]. Subsequent studies on regular ambulances tested a combination of GFAP with clinical scores [83] or GFAP release kinetics and reached negative predictive values of 98.4% [84]. Other, yet smaller, studies could for the first time observe an activation of the neuroinflammatory cascade as early as 36min after symptom onset and could thereby enhance the pathophysiological understanding of ultra-early stroke in humans [85]. First large-scale biomarker studies are being conducted on mobile stroke units to validate other blood-based biomarkers (HFABP and NT-proBNP) for biochemical point-of-care LVO detection onboard regular ambulances [86]. The PROGRESS-BIO study pursues a “multi-omic” screening approach utilizing the advanced capabilities of modern proteomic, metabolomic and genomic techniques to screen for biomarker candidates, which allow for differentiation of ischemic and hemorrhagic stroke as well as stroke-mimicking diseases with the goal of selecting a suitable biomarker panel for prehospital stroke detection within regular ambulances [87]. For other diseases like polytrauma large-scale prehospital biobanks have already been created to facilitate translational and clinical research [88]. A similar approach for acute neurovascular diseases like stroke could have the potential to greatly transform prehospital stroke care. The identification of stroke-specific biomarkers and the consequent development of point-of-care-based test methods could, probably in combination with additional portable devices, telemedicine and artificial intelligence, allow for prehospital stroke identification in normal ambulances in the future. A trajectory like this would have the ability to greatly affect the burden of stroke also in developing and emerging countries with less developed health-systems.

## Conclusion

In addition to their proven beneficial effects in patient care, MSUs are at the forefront of utilizing scientific advancements in the realm of prehospital stroke care. MSUs offer an ideal setting to assess advanced portable devices and biomarkers for stroke detection and thereby promote and accelerate advanced stroke diagnostics for normal ambulances. Collectively, these developments could lead to the adoption of more compact, multimodal methods for the detection and treatment of strokes, potentially revolutionizing future approaches for stroke care on regular ambulances. Such initiatives not only have the potential to transform stroke care in well-developed healthcare systems but also to alleviate the burden of stroke in countries faced with low population density and vast geographic distances as well as those with less resourced medical infrastructures operated solely by regular ambulances. This comprehensive approach to advancing prehospital stroke care underscores the significant role of MSUs in bridging the gap between traditional care models and future innovations in the field.

## Data Availability

No datasets were generated or analysed during the current study.

## Footnotes

1 Berlin Prehospital Or Usual Delivery of Acute Stroke Care [B_PROUD].

2 BEnefits of Stroke Treatment Delivered Using a Mobile Stroke Unit Compared to Standard Management by Emergency Medical Services: The BEST-MSU Study.

## Declarations

All reported studies/experiments with human or animal subjects performed by the authors have been previously published and complied with all applicable ethical standards (including the Helsinki Declaration and its amendments, institutional/national research committee standards, and international/national/institutional guidelines).

## References

[1] GBD 2021 Nervous System Disorders Collaborators. Global, regional, and national burden of disorders affecting the nervous system, 1990-2021: a systematic analysis for the Global Burden of Disease Study 2021. Lancet Neurol. 2024;23(4):344–81. 10.1016/S1474-4422(24)00038-3. 10.1016/S1474-4422(24)00038-3 PMC10949203 38493795

[2] Norrving B, Barrick J, Davalos A, Dichgans M, Cordonnier C, Guekht A (2018). Action plan for stroke in Europe 2018–2030. Eur Stroke J.

[3] Feigin VL, Owolabi MO, World Stroke Organization–Lancet Neurology Commission Stroke Collaboration Group (2023). Pragmatic solutions to reduce the global burden of stroke: a World Stroke Organization-Lancet Neurology Commission. Lancet Neurol.

[4] Fassbender K, Walter S, Liu Y, Muehlhauser F, Ragoschke A, Kuehl S (2003). “Mobile stroke unit” for hyperacute stroke treatment. Stroke.

[5] Ebinger M, Winter B, Wendt M, Weber JE, Waldschmidt C, Rozanski M (2014). Effect of the use of ambulance-based thrombolysis on time to thrombolysis in acute ischemic stroke: a randomized clinical trial. JAMA.

[6] Walter S, Kostopoulos P, Haass A, Keller I, Lesmeister M, Schlechtriemen T (2012). Diagnosis and treatment of patients with stroke in a mobile stroke unit versus in hospital: a randomised controlled trial. Lancet Neurol.

[7] Ebinger M, Kunz A, Wendt M, Rozanski M, Winter B, Waldschmidt C (2015). Effects of golden hour thrombolysis: a Prehospital Acute Neurological Treatment and Optimization of Medical Care in Stroke (PHANTOM-S) substudy. JAMA Neurol.

[8] Wendt M, Ebinger M, Kunz A, Rozanski M, Waldschmidt C, Weber JE (2015). Improved prehospital triage of patients with stroke in a specialized stroke ambulance: results of the pre-hospital acute neurological therapy and optimization of medical care in stroke study. Stroke.

[9] Taqui A, Cerejo R, Itrat A, Briggs FBS, Reimer AP, Winners S (2017). Reduction in time to treatment in prehospital telemedicine evaluation and thrombolysis. Neurology.

[10] Helwig SA, Ragoschke-Schumm A, Schwindling L, Kettner M, Roumia S, Kulikovski J (2019). Prehospital stroke management optimized by use of clinical scoring vs mobile stroke unit for triage of patients with stroke: a randomized clinical trial. JAMA Neurol.

[11] Zhao H, Coote S, Easton D, Langenberg F, Stephenson M, Smith K (2020). Melbourne mobile stroke unit and reperfusion therapy: greater clinical impact of thrombectomy than thrombolysis. Stroke.

[12] Cooley SR, Zhao H, Campbell BCV, Churilov L, Coote S, Easton D (2021). Mobile stroke units facilitate prehospital management of intracerebral hemorrhage. Stroke.

[13] Larsen K, Jaeger HS, Tveit LH, Hov MR, Thorsen K, Røislien J (2021). Ultraearly thrombolysis by an anesthesiologist in a mobile stroke unit: A prospective, controlled intervention study. Eur J Neurol.

[14] Ebinger M, Siegerink B, Kunz A, Wendt M, Weber JE, Schwabauer E (2021). Association between dispatch of mobile stroke units and functional outcomes among patients with acute ischemic stroke in Berlin. JAMA.

[15] Grotta JC, Yamal JM, Parker SA, Rajan SS, Gonzales NR, Jones WJ (2021). Prospective, multicenter, controlled trial of mobile stroke units. N Engl J Med.

[16] Turc G, Hadziahmetovic M, Walter S, Churilov L, Larsen K, Grotta JC (2022). Comparison of mobile stroke unit with usual care for acute ischemic stroke management: a systematic review and meta-analysis. JAMA Neurol.

[17] Mackey J, Yamal JM, Parker SA, Silnes K, Rajan SS, Jacob AP (2023). Golden hour treatment with tPA (Tissue-Type Plasminogen Activator) in the BEST-MSU study. Stroke.

[18] Walter S, Audebert HJ, Katsanos AH, Larsen K, Sacco S, Steiner T (2022). European Stroke Organisation (ESO) guidelines on mobile stroke units for prehospital stroke management. Eur Stroke J.

[19] Lund UH, Stoinska-Schneider A, Larsen K, Bache KG, Robberstad B (2022). Cost-effectiveness of mobile stroke unit care in Norway. Stroke.

[20] Reimer AP, Zafar A, Hustey FM, Kralovic D, Russman AN, Uchino K (2019). Cost-consequence analysis of mobile stroke units vs. standard prehospital care and transport. Front Neurol.

[21] Oliveira Gonçalves AS, Rohmann JL, Piccininni M, Kurth T, Ebinger M, Endres M (2023). Economic evaluation of a mobile stroke unit service in Germany. Ann Neurol.

[22] Kim J, Easton D, Zhao H, Coote S, Sookram G, Smith K (2021). Economic evaluation of the Melbourne mobile stroke unit. Int J Stroke Off J Int Stroke Soc.

[23] Rink JS, Froelich MF, Nour M, Saver JL, Szabo K, Hoyer C, Fassbender KC, Schoenberg SO, Tollens F. Lifetime economic potential of mobile stroke units in acute stroke care: A model-based analysis of the drivers of cost-effectiveness. J Telemed Telecare. 2022;1357633X221140951. 10.1177/1357633X221140951. 10.1177/1357633X221140951 36484406

[24] Chen J, Lin X, Cai Y, Huang R, Yang S, Zhang G (2022). A systematic review of Mobile stroke unit among acute stroke patients: time metrics, adverse events, functional result and cost-effectiveness. Front Neurol.

[25] About PRESTO | PRE-Hospital Stroke Treatment Organization [Internet]. [cited 2024 Mar 25]. Available from: https://www.prestomsu.org/i4a/pages/index.cfm?pageid=3268. **The prehospital stroke treatment organization, an international consortium of medical practitioners involved in pre-hospital treatment of patients with acute stroke**.

[26] Shafqat S, Kvedar JC, Guanci MM, Chang Y, Schwamm LH (1999). Role for telemedicine in acute stroke. Feasibility and reliability of remote administration of the NIH stroke scale. Stroke.

[27] Audebert HJ, Schenkel J, Heuschmann PU, Bogdahn U, Haberl RL, Telemedic Pilot Project for Integrative Stroke Care Group (2006). Effects of the implementation of a telemedical stroke network: the Telemedic Pilot Project for Integrative Stroke Care (TEMPiS) in Bavaria, Germany. Lancet Neurol.

[28] LaMonte MP, Bahouth MN, Hu P, Pathan MY, Yarbrough KL, Gunawardane R (2003). Telemedicine for acute stroke: triumphs and pitfalls. Stroke.

[29] Linderoth G, Lippert F, Østergaard D, Ersbøll AK, Meyhoff CS, Folke F (2021). Live video from bystanders’ smartphones to medical dispatchers in real emergencies. BMC Emerg Med.

[30] Bielski K, Böttiger BW, Pruc M, Gasecka A, Sieminski M, Jaguszewski MJ (2022). Outcomes of audio-instructed and video-instructed dispatcher-assisted cardiopulmonary resuscitation: a systematic review and meta-analysis. Ann Med.

[31] Sýkora R, Peřan D, Renza M, Bradna J, Smetana J, Duška F (2022). Video emergency calls in medical dispatching: a scoping review. Prehospital Disaster Med.

[32] Wyckoff MH, Singletary EM, Soar J, Olasveengen TM, Greif R, Liley HG (2021). 2021 International consensus on cardiopulmonary resuscitation and emergency cardiovascular care science with treatment recommendations. Resuscitation.

[33] Miller M, Bootland D, Jorm L, Gallego B (2022). Improving ambulance dispatch triage to trauma: A scoping review using the framework of development and evaluation of clinical prediction rules. Injury.

[34] Ferri P, Sáez C, Félix-De Castro A, Juan-Albarracín J, Blanes-Selva V, Sánchez-Cuesta P (2021). Deep ensemble multitask classification of emergency medical call incidents combining multimodal data improves emergency medical dispatch. Artif Intell Med.

[35] Tollinton L, Metcalf AM, Velupillai S (2020). Enhancing predictions of patient conveyance using emergency call handler free text notes for unconscious and fainting incidents reported to the London Ambulance Service. Int J Med Inf.

[36] Byrsell F, Claesson A, Ringh M, Svensson L, Jonsson M, Nordberg P (2021). Machine learning can support dispatchers to better and faster recognize out-of-hospital cardiac arrest during emergency calls: A retrospective study. Resuscitation.

[37] Kang DY, Cho KJ, Kwon O, Kwon Jm, Jeon KH, Park H (2020). Artificial intelligence algorithm to predict the need for critical care in prehospital emergency medical services. Scand J Trauma Resusc Emerg Med.

[38] Chin KC, Hsieh TC, Chiang WC, Chien YC, Sun JT, Lin HY (2021). Early recognition of a caller’s emotion in out-of-hospital cardiac arrest dispatching: An artificial intelligence approach. Resuscitation.

[39] Khoirunnisaa K, Hartanto R, Wayan Mustika I, Woraratpanya K, Arva Arshella I. AI-enabled exit strategy of emergency vehicle preemption. In: 2023 15th Int Conf Inf Technol Electr Eng (ICITEE). 2023;99–104. 10.1109/ICITEE59582.2023.10317753.

[40] Kashou AH, Adedinsewo DA, Siontis KC, Noseworthy PA. Artificial intelligence-enabled ECG: Physiologic and pathophysiologic insights and implications. Compr Physiol. 2022;12(3):3417–424. 10.1002/cphy.c210001. 10.1002/cphy.c210001 PMC9795459 35766831

[41] Chee ML, Chee ML, Huang H, Mazzochi K, Taylor K, Wang H (2023). Artificial intelligence and machine learning in prehospital emergency care: A scoping review. iScience.

[42] Chenais G, Lagarde E, Gil-Jardiné C (2023). Artificial intelligence in emergency medicine: viewpoint of current applications and foreseeable opportunities and challenges. J Med Internet Res.

[43] Scholz ML, Collatz-Christensen H, Blomberg SNF, Boebel S, Verhoeven J, Krafft T (2022). Artificial intelligence in Emergency Medical Services dispatching: assessing the potential impact of an automatic speech recognition software on stroke detection taking the Capital Region of Denmark as case in point. Scand J Trauma Resusc Emerg Med.

[44] Le VT, Tran-Trung K, Hoang VT (2022). A comprehensive review of recent deep learning techniques for human activity recognition. Comput Intell Neurosci.

[45] Tillmann JF, Hsu AI, Schwarz MK, Yttri EA (2024). A-SOiD, an active-learning platform for expert-guided, data-efficient discovery of behavior. Nat Methods..

[46] Mayampurath A, Parnianpour Z, Richards CT, Meurer WJ, Lee J, Ankenman B (2021). Improving prehospital stroke diagnosis using natural language processing of paramedic reports. Stroke.

[47] Scott IM, Manoczki C, Swain AH, Ranjan A, McGovern MG, Shyrell Tyson AL (2022). Prehospital telestroke vs paramedic scores to accurately identify stroke reperfusion candidates: a cluster randomized controlled trial. Neurology.

[48] Melaika K, Sveikata L, Vilionskis A, Wiśniewski A, Jurjans K, Klimašauskas A (2022). Prehospital stroke care, paramedic training needs, and hospital-directed feedback in Lithuania. Healthc Basel Switz.

[49] Duvekot MHC, Venema E, Rozeman AD, Moudrous W, Vermeij FH, Biekart M (2021). Comparison of eight prehospital stroke scales to detect intracranial large-vessel occlusion in suspected stroke (PRESTO): a prospective observational study. Lancet Neurol.

[50] Nguyen TTM, van den Wijngaard IR, Bosch J, van Belle E, van Zwet EW, Dofferhoff-Vermeulen T (2021). Comparison of prehospital scales for predicting large anterior vessel occlusion in the ambulance setting. JAMA Neurol.

[51] Cao M, Guan T, Han X, Shen B, Chao B, Liu Y (2021). Impact of a health campaign on Chinese public awareness of stroke: evidence from internet search data. BMJ Open.

[52] Deoni SCL, Medeiros P, Deoni AT, Burton P, Beauchemin J, D’Sa V (2022). Development of a mobile low-field MRI scanner. Sci Rep.

[53] Kimberly WT, Sorby-Adams AJ, Webb AG, Wu EX, Beekman R, Bowry R (2023). Brain imaging with portable low-field MRI. Nat Rev Bioeng.

[54] Roberts DR, McGeorge T, Abrams D, Hewitt R, LeBlanc D, Dennis W (2023). Mobile point-of-care MRI demonstration of a normal volunteer in a telemedicine-equipped ambulance. J Stroke Cerebrovasc Dis Off J Natl Stroke Assoc.

[55] Cowell K, Pang TY, Kwok JS, McCrowe C, Langenberg F, Easton D (2023). Can we miniaturize CT technology for a successful mobile stroke unit roll-out?. Annu Int Conf IEEE Eng Med Biol Soc IEEE Eng Med Biol Soc Annu Int Conf.

[56] Kwok JS, Fox K, Bil C, Langenberg F, Balabanski AH, Dos Santos A (2022). Bringing CT scanners to the skies: design of a CT scanner for an air mobile stroke unit. Appl Sci.

[57] Hubert GJ, Hubert ND, Maegerlein C, Kraus F, Wiestler H, Müller-Barna P (2022). Association between use of a flying intervention team vs patient interhospital transfer and time to endovascular thrombectomy among patients with acute ischemic stroke in nonurban Germany. JAMA.

[58] Guo X, Dye J (2023). Modern prehospital screening technology for emergent neurovascular disorders. Adv Biol.

[59] Siniscalchi A, Malferrari G, Lochner P, Sanguigni S (2021). Transcranial doppler ultrasonography in pre-hospital management of stroke: can it make a difference?. Curr Med Imaging.

[60] Yang R, Wang M, Dong Q, Zhou X (2023). Transcranial Doppler versus CT angiography: a comparative analysis for the diagnosis of ischaemic cerebrovascular disease. Clin Radiol.

[61] Ince J, Banahan C, Venturini S, Alharbi M, Turner P, Oura M (2020). Acute ischemic stroke diagnosis using brain tissue pulsations. J Neurol Sci.

[62] Molaie AM, Wilfling S, Kilic M, Wendl C, Linker RA, Schlachetzki F, et al. Use of the SONAS ultrasound device for the assessment of cerebral perfusion in acute ischemic stroke. Stroke Vasc Interv Neurol. 2024;10(10):e001092. https://www.ahajournals.org/doi/10.1161/SVIN.123.001092. 10.1161/SVIN.123.001092 PMC12778504 41584477

[63] Kilic M, Scalzo F, Lyle C, Baldaranov D, Dirnbacher M, Honda T (2022). A mobile battery-powered brain perfusion ultrasound (BPU) device designed for prehospital stroke diagnosis: correlation to perfusion MRI in healthy volunteers. Neurol Res Pract.

[64] Clare K, Stein A, Damodara N, Feldstein E, Alshammari H, Ali S (2022). Safety and efficacy of a novel robotic transcranial doppler system in subarachnoid hemorrhage. Sci Rep.

[65] Niesen WD, Schlaeger A, Bardutzky J, Fuhrer H (2019). Correct Outcome prognostication via sonographic volumetry in supratentorial intracerebral hemorrhage. Front Neurol.

[66] Shukla D, Konar S, Devi BI, Padmasri G, Jayanna R, Suresh M (2023). Performance of a new portable near-infrared spectroscopy device for detection of traumatic intracranial hematoma. Injury.

[67] Kwon H, Kim K, Jo YH, Park MJ, Ko SB, Kim TJ (2018). Early detection of cerebral infarction with middle cerebral artery occlusion with functional near-infrared spectroscopy: a pilot study. Front Neurol.

[68] Collette SL, Venema AM, Eleveld N, Absalom AR, Scheeren TW, Verhoeve S (2022). Near-infrared spectroscopy monitoring during endovascular treatment for acute ischaemic stroke. Eur Stroke J.

[69] Robertson CS, Zager EL, Narayan RK, Handly N, Sharma A, Hanley DF (2010). Clinical evaluation of a portable near-infrared device for detection of traumatic intracranial hematomas. J Neurotrauma.

[70] Guo L, Abbosh A (2018). Stroke localization and classification using microwave tomography with k-means clustering and support vector machine. Bioelectromagnetics.

[71] Alon L, Dehkharghani S (2021). A stroke detection and discrimination framework using broadband microwave scattering on stochastic models with deep learning. Sci Rep.

[72] Ismail D, Mustafa S (2023). Diagnosis of a brain stroke using wideband microwave scattering. R Soc Open Sci.

[73] Guo L, Khosravi-Farsani M, Stancombe A, Bialkowski K, Abbosh A (2022). Adaptive clustering distorted born iterative method for microwave brain tomography with stroke detection and classification. IEEE Trans Biomed Eng.

[74] Persson M, Fhager A, Trefná HD, Yu Y, McKelvey T, Pegenius G (2014). Microwave-based stroke diagnosis making global prehospital thrombolytic treatment possible. IEEE Trans Biomed Eng.

[75] Candefjord S, Winges J, Malik AA, Yu Y, Rylander T, McKelvey T (2017). Microwave technology for detecting traumatic intracranial bleedings: tests on phantom of subdural hematoma and numerical simulations. Med Biol Eng Comput.

[76] Tsiftsis D, Manioti EA, Touris G, Kyriakakis E, Tsamopoulos N, Gamvroudi M (2024). Detecting stroke at the emergency department by a point of care device: a multicenter feasibility study. Med Devices Auckl NZ.

[77] Kellner CP, Sauvageau E, Snyder KV, Fargen KM, Arthur AS, Turner RD (2018). The VITAL study and overall pooled analysis with the VIPS non-invasive stroke detection device. J Neurointerventional Surg.

[78] Yin L, Yu T, Cheng L, Liu X, Zhang W, Zhang H (2022). Laser speckle contrast imaging for blood flow monitoring in predicting outcomes after cerebral ischemia-reperfusion injury in mice. BMC Neurosci.

[79] Favilla CG, Carter S, Hartl B, Gitlevich R, Mullen MT, Yodh AG (2024). Validation of the Openwater wearable optical system: cerebral hemodynamic monitoring during a breath-hold maneuver. Neurophotonics.

[80] Favilla CG, Baird GL, Grama K, Konecky S, Carter S, Smith W, Gitlevich R, Lebron-Cruz A, Yodh AG, McTaggart RA. Portable cerebral blood flow monitor to detect large vessel occlusion in patients with suspected stroke. J Neurointerv Surg. 2024;1:jnis-2024–021536. 10.1136/jnis-2024-021536. 10.1136/jnis-2024-021536 PMC11415534 38514189

[81] van Stigt MN, Groenendijk EA, van Meenen LCC, van de Munckhof AAGA, Theunissen M, Franschman G (2023). Prehospital detection of large vessel occlusion stroke with EEG. Neurology.

[82] Rozanski M, Waldschmidt C, Kunz A, Grittner U, Ebinger M, Wendt M (2017). Glial fibrillary acidic protein for prehospital diagnosis of intracerebral hemorrhage. Cerebrovasc Dis Basel Switz.

[83] Jæger HS, Tranberg D, Larsen K, Valentin JB, Blauenfeldt RA, Luger S (2023). Diagnostic performance of Glial Fibrillary Acidic Protein and Prehospital Stroke Scale for identification of stroke and stroke subtypes in an unselected patient cohort with symptom onset < 4.5 h. Scand J Trauma Resusc Emerg Med.

[84] Mattila OS, Ashton NJ, Blennow K, Zetterberg H, Harve-Rytsälä H, Pihlasviita S (2021). Ultra-early differential diagnosis of acute cerebral ischemia and hemorrhagic stroke by measuring the prehospital release rate of GFAP. Clin Chem.

[85] Kowalski RG, Ledreux A, Violette JE, Neumann RT, Ornelas D, Yu X (2023). Rapid activation of neuroinflammation in stroke: Plasma and extracellular vesicles obtained on a mobile stroke unit. Stroke.

[86] Kaffes M, Bondi F, Geisler F, Grittner U, Haacke L, Ihl T (2023). Optimization of sensitivity and specificity of a biomarker-based blood test (LVOCheck-Opti): A protocol for a multicenter prospective observational study of patients suspected of having a stroke. Front Neurol.

[87] Geisler F, Haacke L, Lorenz M, Schwabauer E, Wendt M, Bernhardt L (2023). Prospective collection of blood plasma samples to identify potential biomarkers for the prehospital stroke diagnosis (ProGrEss-Bio): study protocol for a multicenter prospective observational study. Front Neurol.

[88] Relja B, Huber-Lang M, van Griensven M, Hildebrand F, Maegele M, Nienaber U (2020). A nationwide fluidics biobank of polytraumatized patients: implemented by the Network “Trauma Research” (NTF) as an expansion to the TraumaRegister DGU® of the German Trauma Society (DGU). Eur J Trauma Emerg Surg.

